# Plasma p-tau217, p-tau181, and Aβ42 predict amyloid PET positivity in cognitively unimpaired adults

**DOI:** 10.1186/s13195-026-02080-x

**Published:** 2026-05-20

**Authors:** Rui Bao, Wanying Shi, Hongbo Bao, Tonghua Zhang, Xueying Li, Wencai Ding

**Affiliations:** 1 The Second Affiliated Hospital of Wannan Medical College, Wuhu, 241000 China; 2 The First Affiliated Hospital, and College of Clinical Medicine of Henan University of Science and Technology, Luoyang, 471003 China; 3 Department of Neurosurgery, Beijing Tiantan Hospital, Capital Medical University, Beijing, 100070 China; 4 Department of Neurology, The First Affiliated Hospital of Dalian Medical University, Dalian, 116011 China; 5 Department of Neurosurgery, Harbin Medical University Cancer Hospital, Harbin, 150001 China

**Keywords:** P-tau217, Alzheimer’s disease, Plasma biomarkers, Cognition unimpaired

## Abstract

**Background:**

Early detection of Alzheimer's disease (AD) pathology in cognitively unimpaired individuals is critical for preclinical intervention. Plasma biomarkers, especially phosphorylated tau217 (p-tau217), are promising predictors of amyloid-β (Aβ) accumulation.

**Methods:**

In this cohort study, we analyzed data from cognitively unimpaired older adults in the A4 and LEARN studies (*n* = 1,407), comprising 452 participants with Aβ positron emission tomography (PET)-negative status and 955 participants with Aβ PET-positive status. We evaluated the accuracy of plasma biomarkers (p-tau217, p-tau181, Aβ42/40 ratio, and others) in predicting Aβ PET positivity using receiver operating characteristic analysis, comparing covariate-adjusted individual biomarker and biomarker-ratio models with a multivariable combined model integrating plasma biomarkers and covariates. (age, sex, apolipoprotein E [APOE] ε4 genotype).

**Results:**

Plasma p-tau217 showed the strongest individual association with Aβ PET status (area under the curve [AUC], 0.85). A combined model integrating p-tau217, p-tau181, Aβ42, age, sex, and APOE ε4 achieved the highest overall discrimination (AUC, 0.87), although the improvement over the covariate-adjusted p-tau217 model was modest.

**Conclusions:**

Plasma p-tau217 showed the strongest individual performance for predicting Aβ PET positivity in cognitively unimpaired older adults. Adding other plasma biomarkers and clinical covariates provided a modest incremental improvement in classification performance. These findings support blood-based prescreening as a potential enrichment approach, while indicating that confirmatory amyloid assessment remains necessary when definitive Aβ status is required.

**Supplementary Information:**

The online version contains supplementary material available at 10.1186/s13195-026-02080-x.

## Background

With disease-modifying therapies (DMTs) now available in clinical practice, the focus of therapeutic intervention is increasingly shifting to the preclinical stage of AD, where Aβ accumulation occurs in the absence of cognitive decline [1]. Early detection and intervention during this phase hold the potential to delay or prevent the onset of AD-related cognitive impairment [2]. However, current gold-standard diagnostic methods, including cerebrospinal fluid (CSF) analysis and PET, remain invasive, costly, and logistically challenging for widespread use in prevention trials. Blood-based biomarkers (BBMs) offer a promising, less invasive alternative for confirming Aβ pathology, providing a scalable approach to identify individuals likely to harbor cerebral Aβ pathology for preclinical trial screening [1, 3–6].

Among BBMs, plasma phosphorylated tau species (e.g., p-tau217 and p-tau181) and the Aβ42/Aβ40 ratio have shown strong correlations with Aβ burden and have been proposed as promising tools for the early detection of AD [7–12]. Previous studies have demonstrated that p-tau217, in particular, correlates strongly with PET and CSF biomarkers, suggesting its potential as an early indicator of AD-related pathophysiology [13–15]. In addition, p-tau181 remains one of the most extensively studied phospho-tau biomarkers in AD and may provide complementary information in multivariable blood-based prediction models. Moreover, p-tau217 has been shown to predict cognitive decline in preclinical populations, making it a promising biomarker for participant selection in clinical trials [14, 16, 17]. These biomarkers are promising, but their diagnostic performance still requires systematic evaluation in preclinical cohorts enriched for amyloid positivity. This is important because thresholds and operating characteristics established in symptomatic populations may not generalize directly to earlier disease stages, where biomarker concentrations are often lower, pathological burden is less advanced, and the intended use is frequently prescreening rather than diagnosis.

To address these gaps, we conducted a cohort study of cognitively unimpaired older adults from the A4/LEARN study to compare the diagnostic accuracy of six plasma biomarkers and biomarker ratios, including p-tau217, p-tau181, and amyloid-related measures, for predicting Aβ PET positivity. Specifically, this study aimed to (1) evaluate the diagnostic performance of individual biomarkers and their ratios; (2) assess associations with continuous Aβ PET burden; and (3) investigate the utility of combining biomarkers with demographic and genetic covariates to improve classification accuracy. These analyses provide critical insights into the potential role of plasma biomarkers in trial screening and enrich for Aβ PET positivity, facilitating the development of more efficient preclinical trial recruitment strategies.

## Methods

### Study participants

This study used data from the A4 Study and the LEARN Study [18], which were designed based on the amyloid hypothesis of AD, positing Aβ accumulation as an early and central pathogenic process. Participants were community-dwelling older adults aged 65–85 years who were cognitively unimpaired at baseline, with no diagnosis of mild cognitive impairment or dementia. Individuals with unstable medical or psychiatric conditions were excluded, while those with stable comorbidities such as hypertension, diabetes, or hyperlipidemia were eligible. All participants provided written informed consent, and each was required to have a study partner familiar with their daily functioning who also consented to participate.

### Amyloid status definition

Baseline cerebral amyloid burden was assessed using ^18^F-florbetapir PET imaging in participants from the A4 and LEARN studies. Image acquisition and processing followed established study-specific procedures. Aβ burden was quantified using a cortical composite standardized uptake value ratio (SUVR) generated with PMOD software, with normalization to cerebellar gray matter [19]. Amyloid status was determined using PET-derived composite SUVR thresholds applied during the A4 screening process. Participants with SUVR values of 1.15 or greater were classified as amyloid positive based on quantitative thresholds alone [20]. For scans with intermediate SUVR values (1.10–1.15), amyloid status was adjudicated based on independent visual assessment by certified nuclear medicine physicians.

### Plasma biomarker measurements

Plasma biomarker measurements were obtained through the A4 multilaboratory biomarker consortium. Aβ40 and Aβ42 measurements were provided by Araclon Biotech. Plasma p-tau217 was measured by Eli Lilly and Company using an electrochemiluminescence immunoassay, and plasma p-tau181, Aβ40, and Aβ42 were also measured by Roche Diagnostics using prototype electrochemiluminescence immunoassays. All assays were conducted according to established laboratory standard operating procedures. Additional methodological details regarding participating laboratories, analytes, and assay platforms are provided in Supplement 1.

### Statistical analysis

All statistical analyses were conducted using R software (version 4.4.1). Missing data were limited for the primary variables included in the analyses. Primary analyses were therefore conducted using complete-case data. As a sensitivity analysis, missing covariate and selected plasma biomarker values were additionally handled using multivariate imputation by chained equations (MICE). Five imputed datasets were generated, with continuous variables imputed using predictive mean matching, binary variables using logistic regression, and multi-category variables using polytomous regression. Subject identifiers were not imputed and were not used as predictors in the imputation model. Amyloid PET status and plasma p-tau217 were not imputed; observations with missing values in these variables were excluded from the corresponding analyses. Imputation-based analyses yielded results that were directionally consistent with the primary complete-case analyses. Demographic and clinical characteristics were compared between the Aβ PET-negative and Aβ PET-positive groups using independent-samples t tests or Mann–Whitney U tests for continuous variables and χ^2^ tests for categorical variables. Plasma biomarker concentrations (Aβ42, Aβ40, p-tau181, and p-tau217) were log-transformed to improve normality before parametric analyses.

For biomarker ratios, the ratios were first calculated using the raw, untransformed concentrations, and the resulting ratio values were subsequently log10-transformed prior to downstream analyses; individual biomarker concentrations were not log-transformed before ratio construction.

Differences in plasma biomarker levels between groups were evaluated using independent-samples t tests for unadjusted comparisons and linear models for adjusted analyses (accounting for age, sex, and APOE ε4 status). Cohen’s d was used to quantify unadjusted between-group effect sizes only and was calculated using log10-transformed data. Adjusted group comparisons were evaluated separately using linear models, and adjusted Cohen’s d values were not calculated. Receiver operating characteristic curves were constructed to assess the ability of individual biomarkers and combined models to discriminate Aβ PET status. To ensure comparability, all models, including those for individual biomarkers and the combined multi-marker panel, were adjusted for age, sex, and APOE ε4 status as covariates. These covariates were selected a priori because of their established associations with amyloid burden and their relevance in trial-screening settings. The combined multivariable model was developed using LASSO regression for feature selection among all candidate plasma biomarkers, biomarker ratios, and the same prespecified covariates. Interaction terms were not prespecified or tested; all multivariable models were based on main effects only. We did not apply separate stepwise, AIC-based, or nested-model selection procedures for covariates, because the goal was to compare biomarkers under a common clinically relevant adjustment framework rather than to optimize a different covariate structure for each model. Area under the curve values with 95% confidence intervals were calculated, and sensitivity, specificity, positive predictive value, negative predictive value, and overall accuracy were estimated at optimal cutoffs determined using the Youden index. Cutoffs were determined by maximizing Youden’s index. For individual biomarkers, cutoffs are presented in their back-transformed original units. For the multivariable Combined Model, rather than creating an additive score, the cutoff was applied to the predicted probability of Aβ PET positivity derived directly from the multivariable logistic regression equation. The optimal diagnostic cutoff for this predicted probability was determined by maximizing Youden’s index on the ROC curve. Comparisons between ROC curves were performed using the DeLong test [21]. The probability of Aβ PET positivity across the range of biomarker values was estimated using generalized linear models with binary Aβ status as the outcome. For visualization, histogram bars and density curves were scaled to the Aβ probability axis (range, 0–1). Associations between continuous plasma biomarker concentrations and cerebral Aβ burden, expressed as SUVR, were evaluated using linear regression adjusted for age, sex, and APOE ε4 status, with the strength of association quantified by the coefficient of determination (R^2^). Model assumptions were assessed by residual diagnostics, including visual inspection of residual-versus-fitted plots and Q-Q plots of residuals (Supplementary Figures S4 and S5). To assess potential departures from linearity, we additionally compared the primary linear models with natural spline-based models in sensitivity analyses. These spline-based analyses were used to evaluate nonlinearity, whereas the linear models were retained as the primary summary approach for comparability and interpretability. All reported *P* values are raw two-sided values; values smaller than 0.001 are reported as *P* < 0.001 throughout the manuscript. Results with *P* < 0.001 were considered statistically significant after correction, whereas those with values between 0.001 and 0.05 were considered nominally significant. Multivariable feature selection was performed using the least absolute shrinkage and selection operator (LASSO), incorporating all candidate plasma biomarkers, including biomarker ratios, along with relevant covariates.

### External replication in HABS-HD

To assess generalizability, we performed an external replication using data from the Health and Aging Brain Study: Health Disparities (HABS-HD). Participants with available plasma biomarkers, amyloid PET, and covariates were included. Biomarkers and derived ratios were processed as in the primary analyses (including log-transformation).Using a harmonized framework, we evaluated (i) differences between Aβ-positive and Aβ-negative individuals, (ii) associations with continuous amyloid PET burden (global SUVR) using linear regression adjusted for age, sex, and APOE ε4 status, and (iii) classification performance for Aβ positivity using covariate-adjusted logistic models with ROC/AUC.

## Results

### Demographic and clinical characteristics

The study included 1407 cognitively unimpaired participants from the A4/LEARN cohort, comprising 452 Aβ PET-negative and 955 Aβ PET-positive individuals, yielding an Aβ PET positivity prevalence of 67.9% (Table 1). The two groups did not differ significantly with respect to sex distribution or years of education. Participants in the Aβ PET-positive group were older (mean age, 71.9 vs 70.4 years) and had a higher prevalence of APOE ε4 carriage (60.4% vs 22.6%); both differences were statistically significant (*P* < 0.001). Plasma concentrations of all measured biomarkers differed significantly between groups (*P* < 0.001). On cognitive testing, participants in the Aβ PET-positive group had lower Preclinical Alzheimer Cognitive Composite scores (*P* < 0.001), whereas lower Mini-Mental State Examination, Free and Cued Selective Reminding Test, and Digit Symbol Coding Test scores were only nominally significant (*P* = 0.027, 0.020, and 0.002, respectively).

**Table 1:** Sample clinical and demographic characteristics in CU individuals

	All cognitively unimpaired A- (*N* = 452)	All cognitively unimpaired A + (*N* = 955)	*P*-value
Sex (F), n (%)	173 (38.3%)	382 (40.0%)	0.575
Age, years	70.40 (4.28)	71.90 (4.86)	< 0.001
Education, years	16.70 (2.65)	16.60 (2.54)	0.482
APOE ε4 carriage, n (%)	102 (22.6%)	577 (60.4%)	< 0.001
**Cognition**			
PACC	−0.63 (2.21)	−1.12 (2.45)	< 0.001
MMSE	28.90 (1.11)	28.80 (1.27)	0.027
FCSRT96	76.70 (5.78)	75.90 (6.26)	0.020
DSC	44.20 (8.51)	42.70 (8.87)	0.002
LMDR	11.80 (3.40)	11.50 (3.40)	0.059
**Biomarker**			
Aβ42	21.400 [0.938,41.200]	18.800 [0.692, 35.700]	< 0.001
Aβ42/40	105.000 [14.200, 231.000]	91.500 [3.300, 166.000]	< 0.001
p-tau181	1.040 [0.338,3.920]	1.390 [0.466, 8.330]	< 0.001
p-tau181/Aβ42	0.049 [0.019, 1.760]	0.078 [0.022, 3.250]	< 0.001
p-tau217	0.138 [0.077,0.707]	0.236 [0.074, 1.560]	< 0.001
p-tau217/Aβ42	0.007 [0.003, 0.203]	0.014 [0.003, 0.465]	< 0.001

*Abbreviations*: *Aβ PET* amyloid-β positron emission tomography, *Aβ, amyloid-β* APOE, apolipoprotein E, *CU* cognitively unimpaired, *DSC*, Digit Symbol Coding Test, *FCSRT* Free and Cued Selective Reminding Test, *LMDR* Logical Memory Delayed Recall, *MMSE* Mini-Mental State Examination, *PACC* Preclinical Alzheimer Cognitive Composite, *PET* positron emission tomography, *p-tau* phosphorylated tau, *SD* standard deviation, *SUVR* standardized uptake value ratio

Amyloid PET positivity was defined according to the A4 screening algorithm: a global SUVR ≥ 1.15, or an SUVR between 1.10 and 1.15 adjudicated by visual assessment. Continuous variables are presented as mean (SD), and categorical variables are presented as number (percentage). Plasma biomarker concentrations are presented as Median (IQR). All reported *P* values are raw two-sided values. Statistical significance after Bonferroni correction was defined as *P* < 0.001; values between 0.001 and 0.05 were considered nominally significant

### Comparison of plasma biomarker profiles by Aβ PET status

**Fig. 1:**
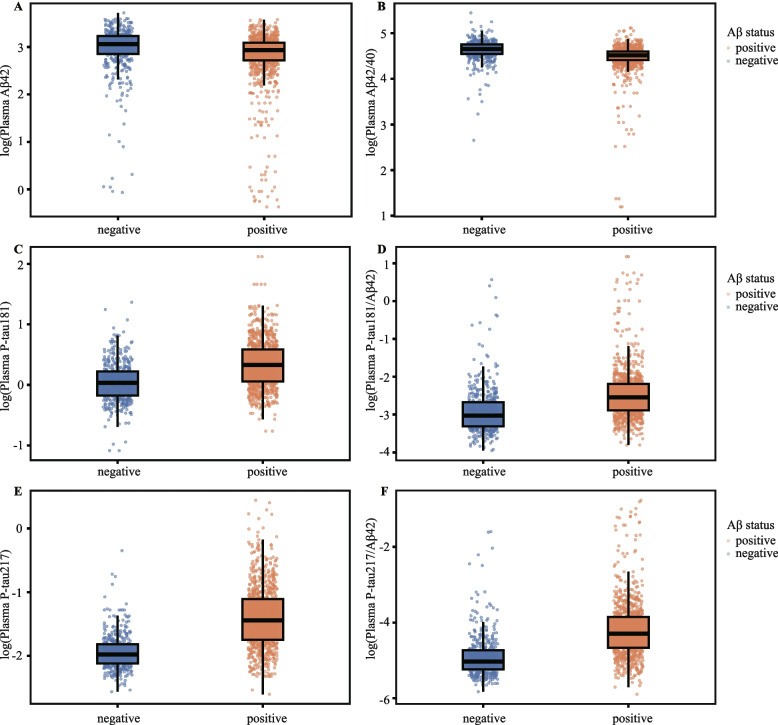
Plasma Biomarker Distributions Stratified by Amyloid-β PET Status**.** Distributions of log-transformed plasma biomarkers are shown according to Aβ PET status. Panels show (**A**) Aβ42, (**B**) Aβ42/40 ratio, (**C**) p-tau181, (**D**) p-tau181/Aβ42 ratio, (**E**) p-tau217, and (**F**) p-tau217/Aβ42 ratio. Each point represents an individual participant. Orange circles indicate Aβ PET–positive individuals, and blue circles indicate Aβ PET–negative individuals. This figure is descriptive and unadjusted; no covariate adjustment was applied. Abbreviations: Aβ, amyloid-β; PET, positron emission tomography; p-tau, phosphorylated tau

The distributions of the six log-transformed plasma biomarkers stratified by Aβ PET status are shown in Fig. 1, and quantitative comparisons between the Aβ PET-negative and Aβ PET-positive groups are summarized in Table 2. All plasma biomarkers differed significantly between groups (*P* < 0.001), with unadjusted effect sizes quantified using Cohen’s d ranging from moderate to large. Phosphorylated tau biomarkers showed the greatest separation between groups. Plasma p-tau217 demonstrated the largest effect size (Cohen’s d = − 1.30), indicating substantially higher levels in Aβ PET-positive individuals. The p-tau217/Aβ42 ratio also showed a large effect size (Cohen’ s d = − 1.02). Among amyloid-related biomarkers, the Aβ42/40 ratio showed a moderate effect size (Cohen’s d = 0.53), whereas Aβ42 exhibited the smallest between-group difference (Cohen’s d = 0.28). Plasma p-tau181 and the p-tau181/Aβ42 ratio showed moderate effect sizes (Cohen’s d = − 0.80 and − 0.69, respectively). These between-group differences remained robust and statistically significant (*P* < 0.001) after adjustment for age, sex, and *APOE* ε4 allele status, with the direction of the differences and the relative magnitude of the effect sizes remaining consistent with the unadjusted findings (Table 2).

**Table 2:** Comparison of plasma biomarker levels between Aβ-PET groups

Biomarker	Mean (SD) PET-Aβ-	Mean (SD) PET-Aβ +	Cohen’s d	*P* value^a^	*P* value^b^
Aβ42	21.0 (6.89)	18.3 (6.15)	0.282	8.91E-7	2.39E-04
Aβ42/40	104 (21.0)	90.2 (18.5)	0.529	7.54E-20	1.76E-11
p-tau181	1.10 (0.401)	1.52 (0.702)	−0.803	3.76E-42	3.60E-27
p-tau181/Aβ42	0.074 (0.133)	0.127 (0.255)	−0.693	2.43E-32	1.59E-19
p-tau217	0.149 (0.053)	0.279 (0.165)	−1.30	2.45E-97	3.37E-67
p-tau217/Aβ42	0.010 (0.017)	0.023 (0.043)	−1.02	7.60E-65	2.97E-42

*Abbreviations*: *Aβ* amyloid-β, *PET* positron emission tomography, *p-tau* phosphorylated tau, *SD* standard deviation; SUVR, standardized uptake value ratio.* P* value^a^: *P* value derived from comparison of means using log-transformed biomarker data without adjustment for age, sex, APOE ε4 allele status. *P* value^b^:* P* value derived from comparison of means using log-transformed biomarker data with adjustment for age, sex, APOE ε4 allele status

Data are presented as mean (SD) for the original, untransformed biomarker values. *P* value^a^ and *P* value^b^ were derived from analyses performed using log-transformed biomarker data; *P* value^a^ represents the unadjusted comparison, and *P* value^b^ represents the comparison adjusted for age, sex, and APOE ε4 allele status. Cohen’s d values represent unadjusted between-group effect sizes only. Adjusted group comparisons were evaluated separately using linear models. All reported *P* values are raw two-sided values. Statistical significance after Bonferroni correction was defined as *P* < 0.001

### Associations between plasma biomarkers and Aβ PET burden

**Fig. 2:**
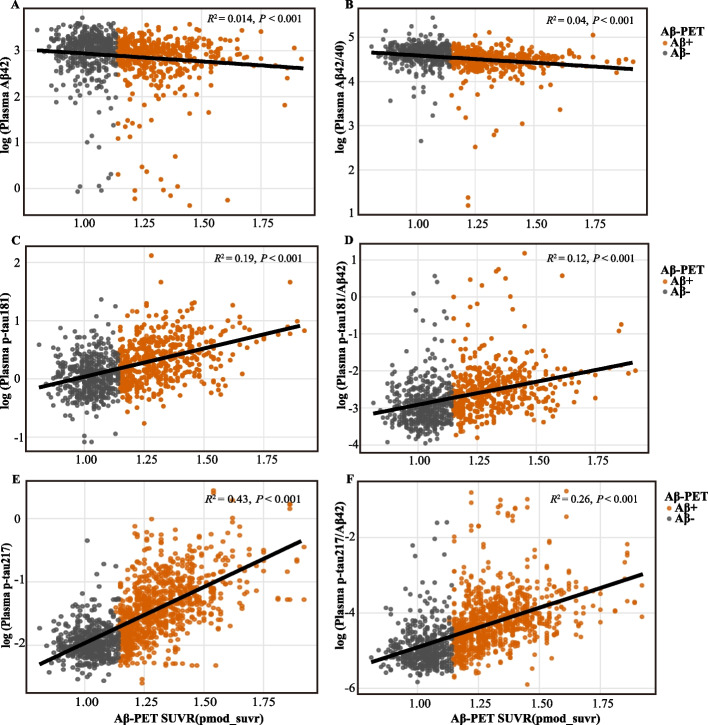
Associations Between Plasma Biomarkers and Amyloid-β PET Burden. Scatterplots show the associations between six log-transformed plasma biomarkers and continuous Aβ PET SUVR. Panels show (**A**) Aβ42, (**B**) Aβ42/40 ratio, (**C**) p-tau181, (**D**) p-tau181/Aβ42 ratio, (**E**) p-tau217, and (**F**) p-tau217/Aβ42 ratio. Black lines indicate linear regression fits from models including the biomarker of interest plus age, sex, and APOE ε4 allele status as covariates, with the coefficient of determination (R^2^) and corresponding *P* values shown for each panel. Orange dots represent Aβ PET–positive participants, whereas gray dots represent Aβ PET–negative participants. Abbreviations: Aβ, amyloid-β; PET, positron emission tomography; p-tau, phosphorylated tau; SUVR, standardized uptake value ratio

**Fig. 3:**
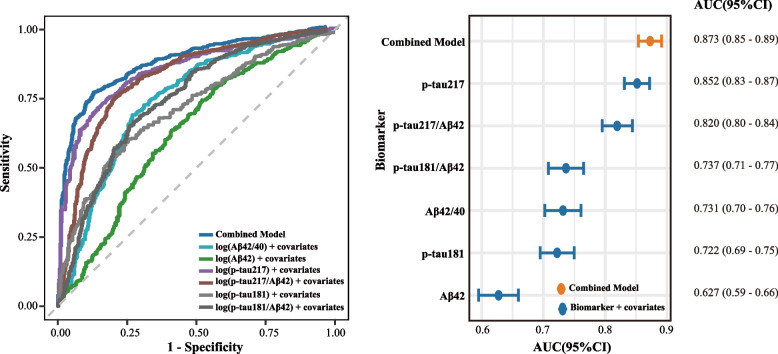
Receiver Operating Characteristic Curves for Covariate-Adjusted Plasma Biomarker Models and the Combined Model. Receiver operating characteristic curves are shown for covariate-adjusted individual biomarker and biomarker-ratio models and for the combined model for predicting Aβ PET positivity. All ROC models were adjusted for age, sex, and APOE ε4 allele status. Individual curves correspond to the following predictor sets: log(Aβ42/Aβ40) + age + sex + APOE ε4; log(p-tau217/Aβ42) + age + sex + APOE ε4; log(Aβ42) + age + sex + APOE ε4; log(p-tau217) + age + sex + APOE ε4; log(p-tau181) + age + sex + APOE ε4; and log(p-tau181/Aβ42) + age + sex + APOE ε4. The combined model included log(p-tau217), log(p-tau181), log(Aβ42), age, sex, and APOE ε4 allele status. Abbreviations: Aβ, amyloid-β; APOE, apolipoprotein E; p-tau, phosphorylated tau; ROC, Receiver Operating Characteristic Curves

We next examined associations between log-transformed plasma biomarker concentrations and continuous Aβ PET burden, quantified using SUVR, with the strength of association assessed by linear regression (Fig. 2). Among all biomarkers, plasma p-tau217 explained the greatest proportion of variance in global Aβ PET burden (R^2^ = 0.43; *P* < 0.001). The p-tau217/Aβ42 ratio also showed a relatively strong association (R^2^ = 0.26; *P* < 0.001). In contrast, plasma p-tau181 and the p-tau181/Aβ42 ratio showed more modest associations with Aβ PET SUVR (*R*^*2*^ = 0.19 and 0.12, respectively; both *P* < 0.001). Amyloid-related plasma biomarkers showed weaker associations with cerebral Aβ burden. The Aβ42/40 ratio and Aβ42 were associated with Aβ PET SUVR with low coefficients of determination (*R*^*2*^ = 0.04 and 0.014, respectively), despite reaching statistical significance (both *P* < 0.001). Classification performance, including sensitivity, specificity, positive predictive value, and negative predictive value, is summarized in Fig. 3 and Table S1. Plasma p-tau217 and the p-tau217/Aβ42 ratio were associated with lower rates of false-positive and false-negative classifications compared with other biomarkers, whereas p-tau181-based measures showed higher false-negative rates. Amyloid-based plasma measures showed limited discriminative performance, with higher false-positive rates observed for the Aβ42/40 ratio. From a screening perspective, these patterns may have practical implications because false positives could increase the number of individuals referred for confirmatory PET or CSF testing, whereas false negatives could miss individuals with underlying amyloid pathology who might otherwise proceed to further evaluation or prevention-trial prescreening. Consistent with this pattern, p-tau217-based measures showed a lower overall burden of false-positive and false-negative classifications than biomarkers associated with comparatively higher false-positive or false-negative rates. In supplementary sensitivity analyses, spline-based models suggested evidence of nonlinearity for most biomarkers, including Aβ42/Aβ40, p-tau217/Aβ42, Aβ42, p-tau217, and p-tau181/Aβ42 (all *P* < 0.001), whereas p-tau181 showed only nominal evidence of nonlinearity (*P* = 0.006; Supplementary Table S3).

### Performance of plasma biomarkers for predicting Aβ status

The diagnostic performance of individual log-transformed plasma biomarkers, biomarker ratios, and a combined model for classifying Aβ PET status was evaluated using receiver operating characteristic analysis, with all models adjusted for the same a priori covariates: age, sex, and APOE ε4 allele status. The individual biomarker and biomarker-ratio models were prespecified single-biomarker models, each adjusted for age, sex, and APOE ε4 allele status. To identify the optimal multivariable panel, we applied LASSO regression for feature selection among all candidate plasma biomarkers, biomarker ratios, and covariates. The LASSO-selected combined model retained p-tau217, p-tau181, Aβ42, age, sex, and APOE ε4 allele status, and showed the highest discriminative performance (AUC, 0.87; 95% CI, 0.85–0.89). Compared with the covariate-adjusted plasma p-tau217 model, the improvement in AUC of the combined model was modest and did not reach statistical significance (DeLong’s test, *P* = 0.083; Supplementary Table S2). Calibration plots for the combined model and the covariate-adjusted plasma p-tau217 model are provided in Supplementary Figure S3. Among individual biomarkers and ratios, plasma p-tau217 demonstrated the strongest performance (AUC, 0.85; 95% CI, 0.83–0.87), followed by the p-tau217/Aβ42 ratio (AUC, 0.82; 95% CI, 0.80–0.84). The Aβ42/40 ratio showed moderate discriminative ability (AUC, 0.73; 95% CI, 0.70–0.76). The performance of p-tau181/Aβ42 and p-tau181 were similar (AUC, 0.74; 95% CI, 0.71–0.77 and AUC, 0.72; 95% CI, 0.69–0.75, respectively). Plasma Aβ42 showed the lowest discriminative performance (AUC, 0.63; 95% CI, 0.59–0.66). Overall, combining plasma p-tau217 with amyloid-related biomarkers and clinical covariates improved the classification of Aβ PET status compared with individual biomarkers. The resulting combined model, which included p-tau217, p-tau181, and Aβ42, age, sex, and APOE ε4 allele status, showed the highest discriminative performance (AUC, 0.87; 95% CI, 0.85–0.89).

### Accuracy and classification metrics of plasma biomarkers using single cutoffs

Using optimal cutoffs derived from the Youden index, we evaluated the diagnostic performance of individual log-transformed plasma biomarkers and their ratios for predicting Aβ PET positivity in cognitively unimpaired older adults (Table S1 and Figure S1). The p-tau217/Aβ42 ratio showed the highest accuracy (76.69%) and a balanced sensitivity and specificity profile (76.34% and 77.43%, respectively), followed by plasma p-tau217 (accuracy, 76.26%; specificity, 85.84%). Other biomarkers, including the Aβ42/40 ratio and the p-tau181/Aβ42 ratio, showed moderate accuracy (69.58% and 68.16%, respectively). Plasma Aβ42 showed the lowest accuracy (66.10%) and specificity (46.46%). A combined model incorporating p-tau217, p-tau181, and Aβ42, along with age, sex, and APOE ε4 allele status, showed improved overall performance (accuracy, 80.03%; AUC, 0.87), with higher specificity (86.28%) and positive predictive value (92.23%) than any individual biomarker or ratio. Overall, plasma p-tau217 was the strongest single marker, whereas the combined model offered only a modest incremental improvement in discrimination.

### External replication in the HABS-HD cohort

Results in HABS-HD were directionally consistent with those in A4/LEARN. Plasma p-tau217 and its ratios showed the strongest associations with amyloid pathology, whereas Aβ-based measures were comparatively weaker. In models of continuous PET burden (SUVR), p-tau217 demonstrated the highest explanatory power, followed by the p-tau217/Aβ42 ratio. In classification analyses, p-tau217-based models provided the best discrimination among individual biomarkers, and the combined model achieved the highest overall performance (Supplementary Materials).

## Discussion

The main results of this cohort study suggest that p-tau217-based plasma measures outperform other plasma biomarkers for identifying ^18^F-florbetapir Aβ PET positivity in cognitively unimpaired older adults. We compared associations with continuous Aβ PET SUVR and classification performance across 6 plasma biomarkers and biomarker ratios. First, p-tau217 showed the strongest association with global Aβ PET burden, and p-tau217 and the p-tau217/Aβ42 ratio showed the largest between-group differences. Second, p-tau217-based measures provided the highest discrimination of Aβ PET positivity; however, negative predictive values were modest in this screening-enriched preclinical sample, limiting rule-out utility when used in isolation. Third, a combined model integrating p-tau217 with p-tau181 and Aβ42, together with age, sex, and APOE ε4 status, provided the best overall classification performance. However, the improvement over the covariate-adjusted plasma p-tau217 model was modest. Accordingly, the potential utility of the combined model should be weighed against its added complexity and the need for external validation before implementation in trial-screening workflows.

Although the overall observation that plasma p-tau217 performs strongly for identifying cerebral amyloid pathology is consistent with prior studies, the present work extends the literature in several clinically and translationally relevant ways. First, our analysis was conducted in the A4/LEARN cohort, a large, well-characterized sample of cognitively unimpaired older adults recruited in a prevention-trial screening context, thereby addressing biomarker performance specifically in a preclinical and screening-enriched setting rather than across mixed clinical stages. Second, we used a unified head-to-head framework to compare multiple plasma biomarkers and biomarker ratios not only for dichotomous Aβ PET classification but also for association with continuous amyloid burden. Third, by directly contrasting covariate-adjusted single-marker models with a LASSO-selected multivariable panel, we show that the principal translational value of this study lies not simply in confirming the strength of p-tau217, but in demonstrating that the additional gain from a more complex combined model is relatively modest in this preclinical trial-enrichment setting. Together, these findings help refine how blood-based biomarkers may be positioned in prevention-oriented screening workflows.

A key implication of these findings is that p-tau217-based measures may capture early downstream responses to cerebral amyloid accumulation more reliably than plasma amyloid peptide measures in cognitively unimpaired individuals [8, 9]. Prior work has consistently shown that p-tau217 performs strongly across platforms and clinical stages, supporting its value as an early indicator of Alzheimer disease-related pathophysiology [7, 22]. In a screening-enriched cohort such as A4/LEARN, plasma Aβ measures may show a narrower dynamic range and may be more sensitive to preanalytical and assay-related variation, which could attenuate their discrimination when amyloid burden is modest and participants remain clinically normal [23]. By contrast, p-tau217 may reflect amyloid-associated tau phosphorylation that becomes detectable even when cognitive performance is largely preserved, thereby yielding a clearer blood-based signal aligned with Aβ PET [8, 24]. This interpretation helps reconcile why p-tau217 outperformed Aβ42 and Aβ42/40 in our cohort while remaining consistent with studies that reported stronger amyloid-peptide performance in samples spanning broader disease severity [23]. The retention of Aβ42, rather than the Aβ42/Aβ40 ratio, in the final combined model should be interpreted as an empirical result of feature selection in this specific screening-enriched cohort, rather than as evidence that Aβ42 is universally preferable across populations or assay settings. Together, these data support using p-tau217-centered plasma profiles as a practical first-line enrichment tool in preclinical trial screening while acknowledging that confirmatory amyloid assessment remains necessary when the goal is to definitively establish Aβ status.

In practical terms, our findings support a stepwise biomarkers-alone blood-first prescreening approach for prevention-oriented pipelines, in which p-tau217-centered measures (alone or combined with complementary markers and readily available covariates) are used to enrich for Aβ PET positivity before confirmatory testing [25]. This framing aligns with recent recommendations that blood-based biomarkers may have their greatest near-term impact when deployed to reduce the number of expensive or less accessible confirmatory procedures, rather than as stand-alone diagnostic substitutes in asymptomatic populations [26, 27]. Importantly, the modest negative predictive values observed in our cohort should be interpreted in the context of the A4/LEARN screening design, where the prevalence of Aβ positivity is high (67.9%) and inevitably constrains rule-out performance even for otherwise strong assays; accordingly, single-marker strategies may be better suited for enrichment (rule-in) than for excluding amyloid pathology in such settings [9, 28, 29]. These misclassification patterns further help explain the practical implications of screening performance in this cohort. False positives may reduce efficiency by increasing the number of individuals referred for unnecessary confirmatory PET or CSF testing, whereas false negatives may lead to missed identification of truly Aβ-positive individuals who might otherwise proceed to further evaluation or trial enrollment. In this context, the more favorable balance of false positives and false negatives observed for p-tau217-based measures supports their use as pragmatic enrichment tools in screening-oriented workflows. This tradeoff was also evident in our study, in which the combined model provided only a modest improvement over the covariate-adjusted p-tau217 model, underscoring that incremental gains in discrimination should be weighed against additional implementation burden in real-world trial-screening workflows.

One practical way to address this limitation is to prespecify operating points tailored to the intended use case, including dual-threshold algorithms that create an intermediate “gray zone” requiring confirmatory PET (or CSF), while allowing high-confidence rule-in and rule-out decisions at the extremes. Recent work evaluating combined p-tau217 and amyloid measures on fully automated platforms demonstrates that dual cutoffs can yield high overall accuracy while leaving a manageable minority of participants in the intermediate zone, providing a concrete template for implementation and triage workflow [7, 30, 31]. Finally, translation will depend on analytical robustness and portability: accumulating evidence from head-to-head comparisons, cut-point optimization studies, and implementation frameworks indicates that assay design and platform-specific calibration can meaningfully influence performance estimates and threshold transferability, reinforcing the need for external validation of any proposed prescreening algorithm across laboratories and more diverse populations before clinical deployment [14, 24, 32, 33].

Despite the robust diagnostic performance of plasma biomarkers observed in this study, their integration into routine clinical practice requires careful consideration of current consensus and guidelines. Currently, prominent organizations such as the Alzheimer’s Association do not recommend the routine use of plasma biomarkers for screening asymptomatic populations in community settings. Current limitations include the uncertain prognostic value of biomarker positivity in asymptomatic individuals and the lack of evidence that existing anti-amyloid therapies provide clinical benefit in asymptomatic individuals. Consequently, identifying Alzheimer’s disease pathology in cognitively unimpaired individuals may not yet substantially alter routine clinical management, although this could evolve as preventive therapeutic strategies and longitudinal prognostic evidence continue to develop. Nevertheless, our results suggest that, in the context of clinical trial enrichment and future prevention-oriented frameworks, blood-based biomarkers may offer a scalable and cost-effective approach to identify individuals more likely to harbor preclinical AD pathology. As therapeutic options expand, these findings may help inform the evidence base for future guideline refinement regarding the use of blood-based tests in early-stage populations.

From a practical perspective, our findings suggest that the highest-performing model is not necessarily the most efficient option for real-world screening workflows. Although the combined model achieved the best overall discrimination, the improvement over the covariate-adjusted plasma p-tau217 model was modest and did not reach statistical significance, indicating that the additional value of incorporating multiple biomarkers may be limited relative to the increased complexity required for implementation. In particular, the combined model included biomarkers measured across different assay platforms, which may increase cost, reduce portability, and complicate standardization in routine prescreening settings. In this context, plasma p-tau217 may offer the most pragmatic balance between diagnostic performance, scalability, and operational simplicity as a first-line blood-based prescreening tool. By contrast, more complex multi-marker models may be most appropriate in settings where maximal enrichment is prioritized, such as prevention trials with limited confirmatory PET capacity or recruitment strategies aiming to minimize unnecessary downstream testing. Accordingly, our findings support a tiered screening framework in which plasma p-tau217 serves as the primary enrichment test, whereas multi-marker panels may be reserved for settings in which the modest incremental gain in classification performance justifies the added assay and logistical burden.

### Limitations

This study has several limitations. First, the cross-sectional design limits causal inference and precludes evaluation of how plasma biomarkers predict longitudinal amyloid accumulation or subsequent cognitive change; prospective follow-up will be needed to establish prognostic utility. Second, the A4/LEARN cohort comprises highly screened, generally healthy volunteers and is predominantly White, which may limit generalizability to more clinically and demographically diverse populations and to settings with lower amyloid prevalence. Third, we defined classification performance using a single “optimal” cutoff derived from the Youden index. Although this approach provides a consistent benchmark for head-to-head comparisons, it may not reflect the operating points most relevant for trial prescreening, where investigators often prioritize either sensitivity (to minimize missed Aβ positivity) or specificity (to reduce confirmatory scans). Relatedly, negative predictive values were modest in this enrichment cohort, and misclassification may be more common among individuals near the PET threshold. Fourth, plasma biomarkers were measured across different laboratories and assay platforms, and although each followed validated procedures, interplatform differences and preanalytical variability may affect absolute values and threshold portability. Finally, the combined model was developed and evaluated within the same cohort; model performance may therefore be optimistic, and external validation is required to confirm generalizability and the stability of coefficients and cut points before implementation in other trial pipelines. In addition, the combined model incorporated biomarkers measured across different assay platforms, which may limit immediate clinical feasibility and further reinforces the need for external validation and platform-harmonized implementation strategies before practical deployment.

## Conclusion

In this cohort study of cognitively unimpaired older adults screened in A4/LEARN, p-tau217-based plasma measures showed the most consistent performance for identifying ^18^F-florbetapir Aβ PET positivity, and a multivariable model that integrated p-tau217 with p-tau181, Aβ42, and key covariates further improved classification. These findings support blood-based prescreening as an enrichment tool for prevention-trial recruitment, while underscoring that confirmatory assessment remains necessary when definitive amyloid status is required. Future work should validate these prescreening algorithms in independent and more diverse cohorts, evaluate prespecified operating points (including potential dual-threshold strategies), and determine longitudinal and real-world screening performance in prevention-oriented trial workflows.

## Supplementary Information

Supplementary Material 1.

Supplementary Material 2.

## Data Availability

All data supporting the findings of this study are available within the paper and its Supplementary Information.

## Code availability

The analysis code used in this study is publicly available on GitHub at: https://github.com/dingneurology/a4learn-plasma-amyloid-analysis.
