# Supplementary material for: Plasma p-tau217, p-tau181, and Aβ42 predict amyloid PET positivity in cognitively unimpaired adults
Source: Alzheimers Res Ther. 2026 May 20;18:136. doi: 10.1186/s13195-026-02080-x (PMC13227887; doi:10.1186/s13195-026-02080-x)
Supplement: Supplementary file 1 — Supplementary Material 1. [file 13195_2026_2080_MOESM1_ESM.docx]

**Plasma Phosphorylated Tau 217, Plasma Phosphorylated Tau 181 and Aβ42 to Predict Early Brain Aβ Accumulation in People Without Cognitive Impairment**

Supplementary Materials

Rui Bao, MBBS^*,1^; Hongbo Bao, MD, PhD*^,2,5^; Zhenzhen Yan, MD, PhD*^,3^; Tonghua Zhang, MD, PhD^*,1^; Xueying Li, MD, PhD^*,4^; Wencai Ding, MD, PhD^*,1^; for the A4 and LEARN Study

*Corresponding Author:

Wencai Ding, MD, PhD

The Second Affiliated Hospital of Wannan Medical College Wuhu, 241000, China

E-mail: [dingneurology@gmail.com](mailto:dingneurology@gmail.com)

Xueying Li, MD, PhD

Department of Neurology, The First Affiliated Hospital of Dalian Medical University, Dalian, 116011, China.

E-mail: [xueyingli0616@163.com](mailto:xueyingli0616@163.com)

Tonghua Zhang, MD, PhD

The Second Affiliated Hospital of Wannan Medical College Wuhu, 241000, China

E-mail: [zth7607@163.com](mailto:zth7607@163.com)

Affiliations:

1. The Second Affiliated Hospital of Wannan Medical College, Wuhu, 241000, China

Department of Neurosurgery, Beijing Tiantan Hospital, Capital Medical University, Beijing, 100070, China.

The fourth affiliated hospital zhejiang university school of medicine, Jinhua, 322023, China

Department of Neurology, The First Affiliated Hospital of Dalian Medical University, Dalian, 116011, China.

Department of Neurosurgery, Harbin Medical University Cancer Hospital, Harbin, 150001, China

**eMethods**

**Cognition**

The Preclinical Alzheimer’s Cognitive Composite (PACC) was the primary efficacy end point of the A4 trial^1,2^. All participants underwent a comprehensive neuropsychological assessment that included the PACC^3^, a composite measure integrating performance across 4 cognitive domains: global cognitive status (Mini-Mental State Examination)^4^, episodic memory (Free and Cued Selective Reminding Test total score)^5^, processing speed and executive function (Digit Symbol Coding Test)^6^, and verbal memory (Logical Memory IIa subtest of the Wechsler Memory Scale)^7,8^. Each component score was standardized to z scores based on the baseline distribution, and the total PACC score was calculated as the sum of these standardized scores. Eligibility criteria included a global Clinical Dementia Rating score of 0, Mini-Mental State Examination scores ranging from 25 to 30, and Wechsler Memory Scale–Revised Logical Memory Delayed Recall scores between 6 and 18.

**Plasma biomarker measurements**
Plasma biomarker measurements were obtained through the A4 multilaboratory biomarker consortium. Plasma samples were shared with external biotechnology companies participating in biomarker assay development and testing. Aβ40 and Aβ42 peptides were quantified by Araclon Biotech, which provided measurements of free, total, and bound plasma Aβ fractions as well as derived ratios. Plasma p-tau217 was measured by Eli Lilly and Company using an electrochemiluminescent immunoassay, with sample preparation automated on a Tecan Fluent workstation and detection performed on an MSD Sector S Imager 600MM. Plasma p-tau181, Aβ40, and Aβ42 were also measured by Roche Diagnostics using Elecsys Robust Prototype electrochemiluminescence immunoassays. The present study used these biomarker values as provided through the A4/LEARN biomarker datasets for secondary analysis. [S1]

**eReferences**

1. Sperling RA, Donohue MC, Raman R, et al. Trial of Solanezumab in Preclinical Alzheimer's Disease. *N Engl J Med*. Sep 21 2023;389(12):1096-1107. doi:10.1056/NEJMoa2305032

2. Donohue MC, Sperling RA, Salmon DP, et al. The preclinical Alzheimer cognitive composite: measuring amyloid-related decline. *JAMA Neurol*. Aug 2014;71(8):961-70. doi:10.1001/jamaneurol.2014.803

3. Sperling RA, Donohue MC, Raman R, et al. Association of Factors With Elevated Amyloid Burden in Clinically Normal Older Individuals. *JAMA Neurol*. Jun 1 2020;77(6):735-745. doi:10.1001/jamaneurol.2020.0387

4. Folstein MF, Folstein SE, McHugh PR. "Mini-mental state". A practical method for grading the cognitive state of patients for the clinician. *J Psychiatr Res*. Nov 1975;12(3):189-98. doi:10.1016/0022-3956(75)90026-6

5. Grober E, Hall CB, Lipton RB, Zonderman AB, Resnick SM, Kawas C. Memory impairment, executive dysfunction, and intellectual decline in preclinical Alzheimer's disease. *J Int Neuropsychol Soc*. Mar 2008;14(2):266-78. doi:10.1017/s1355617708080302

6. Wechsler D, Scale-Revised WAI. The psychological corporation. *San Antonio, TX*. 1997;1:2011-2014.

7. Wechsler D. WAIS-3. WMS-3: Wechsler adult intelligence scale, Wechsler memory scale: Technical manual. 1997;

8. Morris J, Swier-Vosnos A, Woodworth C, Umfleet LG, Czipri S, Kopald B. Development of alternate paragraphs for the Logical Memory subtest of the Wechsler Memory Scale-IV. *Appl Neuropsychol Adult*. 2014;21(2):143-7. doi:10.1080/09084282.2013.780172

S1. A4/LEARN Biomarker Assay Results Methods. A4 Study Data Discovery Portal; accompanying biomarker assay documentation for biomarker_AB_Test.csv, biomarker_pTau217.csv, and biomarker_Plasma_Roche_Results.csv. Accessed November 15, 2025.

**Table S1. Receiver Operating Characteristic Statistics for Individual Plasma Biomarkers, Biomarker Ratios, and the Combined Model**

| Biomarker | AUC  (95% CI) | Cut-off | | Sensitivity  (95% CI) | Specificity  (95% CI) | PPV  (95% CI) | NPV  (95% CI) | Accuracy  (95% CI) |
| --- | --- | --- | --- | --- | --- | --- | --- | --- |
| Aβ42 | 0.63  (0.60 - 0.66) | | 21.99 | 75.39  (56.08 - 83.82) | 46.46  (37.73 - 66.17) | 74.84  (72.51 - 79.20) | 47.19  (40.23 - 54.99) | 66.10  (59.20 - 70.22) |
| Aβ42/40 | 0.73  (0.70 - 0.76) | | 96.54 | 68.80  (66.31 - 78.42) | 71.24  (60.87 - 75.06) | 83.48  (79.95 - 85.84) | 51.94  (48.71 - 58.69) | 69.58  (67.73 - 73.63) |
| p-tau181 | 0.72  (0.69 - 0.75) | | 1.25 | 60.84  (51.26 - 67.05) | 74.78  (68.19 - 84.36) | 83.60  (80.73 - 87.94) | 47.47  (43.23 - 51.61) | 65.32  (61.12 - 68.44) |
| p-tau181/Aβ42 | 0.74  (0.71 - 0.77) | | 0.06 | 66.81  (59.20 - 84.83) | 71.02  (52.38 - 77.98) | 82.96  (78.87 - 86.08) | 50.31  (46.52 - 61.96) | 68.16  (65.16 - 74.35) |
| p-tau217 | 0.85  (0.83 - 0.87) | | 0.18 | 71.73  (66.01 - 82.68) | 85.84  (75.17 - 91.67) | 91.46  (87.31 - 94.37) | 58.97  (54.93 - 68.31) | 76.26  (73.62 - 81.02) |
| p-tau217/Aβ42 | 0.82  (0.79 - 0.84) | | 0.01 | 76.34  (71.90 - 82.87) | 77.43  (70.77 - 82.85) | 87.73  (85.18 - 90.54) | 60.76  (56.34 - 67.04) | 76.69  (74.25 - 79.60) |
| Combined Model | 0.87  (0.85 - 0.89) | | 0.69 | 77.07  (71.11 - 79.80) | 86.28  (83.56 - 91.79) | 92.23  (90.62 - 94.99) | 64.04  (58.84 - 67.85) | 80.03  (77.11 - 82.09) |

Note: Cutoffs were determined by maximizing the Youden index. Statistical analyses were performed using log10-transformed biomarker variables and ratios. For biomarker ratios, ratios were calculated from raw, untransformed concentrations and then log10-transformed. Cutoff values for individual biomarkers and ratios are presented in back-transformed original units for interpretability, whereas the combined model cutoff represents the optimal predicted probability threshold derived from the multivariable logistic regression model. The combined model included p-tau217, p-tau181, and Aβ42; all models included age, sex, and APOE ε4 allele status as covariates.

Abbreviations: Aβ, amyloid-β; AUC, area under the curve; CI, confidence interval; NPV, negative predictive value; PPV, positive predictive value; p-tau, phosphorylated tau.

**Table S2. DeLong test comparisons between the combined model and covariate-adjusted individual biomarker models**

| Biomarker model | Predictor set | AUC | Combined model AUC | DeLong test  *P* value |  |
| --- | --- | --- | --- | --- | --- |
| Aβ42 model | log(Aβ42)+ age + sex + APOE ε4 | 0.754 | 0.873 | <0.001 |  |
| Aβ42/Aβ40 ratio model | log(Aβ42/40) + age + sex + APOE ε4 | 0.787 | 0.873 | <0.001 |  |
| p-tau181 model | log(p-tau181) + age + sex + APOE ε4 | 0.800 | 0.873 | <0.001 |  |
| p-tau181/Aβ42 ratio model | log(p-tau181/ Aβ42)+ age + sex + APOE ε4 | 0.793 | 0.873 | <0.001 |  |
| p-tau217 model | log(p-tau217) + age + sex + APOE ε4 | 0.869 | 0.873 | 0.083 |  |
| p-tau217/Aβ42 ratio model | log(p-tau217/Aβ42) + age + sex + APOE ε4 | 0.841 | 0.873 | <0.001 |  |

| Biomarker | Linear model  AIC | Spline model  AIC | Linear model  Adjusted R² | Spline model  Adjusted R² | P value for nonlinearity |
| --- | --- | --- | --- | --- | --- |
| log(Aβ42) | -1053.1 | -1072.76 | 0.184 | 0.197 | <0.001 |
| log(Aβ42/40) | -1067.51 | -1119.82 | 0.193 | 0.223 | <0.001 |
| log(p-tau181) | -1242.1 | -1248.37 | 0.287 | 0.291 | 0.006 |
| log(p-tau181/ Aβ42) | -1154.85 | -1210.55 | 0.241 | 0.272 | <0.001 |
| log(p-tau217) | -1642.49 | -1672.1 | 0.464 | 0.475 | <0.001 |
| log(p-tau217/ Aβ42) | -1348.29 | -1467.17 | 0.339 | 0.393 | <0.001 |

**Table S3. Comparison of linear and natural spline-based models for associations between plasma biomarkers and continuous Aβ PET SUVR.**

Note: Linear and spline-based models were compared using analysis of variance. Reported P values correspond to tests for nonlinearity. AIC and adjusted R² are shown for descriptive comparison of model fit.

|  | **Aβ- (N=354)** | **Aβ+ (N=96)** | **P-value** |  |
| --- | --- | --- | --- | --- |
| Sex (F), n (%) | 233 (65.8%) | 60 (62.5%) | 0.628 |  |
| Age, years | 66.30 (8.83) | 72.90 (6.85) | <0.001 |  |
| Education, years | 13.10 (4.25) | 13.60 (4.72) | 0.356 |  |
| APOE ε4 carriage, n (%) | 68 (19.2%) | 44 (45.8%) | <0.001 |  |
| Biomarker |  |  |  |  |
| Aβ42 | 7.860 [3.500, 26.800] | 7.180 [1.930, 14.400] | <0.001 |  |
| Aβ42/40 | 0.037 [0.016, 0.064] | 0.032 [0.008, 0.049] | <0.001 |  |
| p-tau181 | 17.700 [4.290, 97.700] | 25.100 [9.130, 71.800] | <0.001 |  |
| p-tau181/Aβ42 | 2.190 [0.590, 23.500] | 3.580 [0.984, 26.700] | <0.001 |  |
| p-tau217 | 0.281 [0.097, 1.44] | 0.684 [0.193, 4.590] | <0.001 |  |
| p-tau217/Aβ42 | 0.036 [0.012, 0.177] | 0.101 [0.018, 1.480] | <0.001 |  |

**Table S4. Baseline demographic and clinical characteristics of participants in the HABS-HD cohort**

**Figure S1. Density plots show log-transformed plasma biomarker values in relation to the predicted probability of Aβ PET positivity (Aβ+).** Panels show Aβ42, Aβ42/40 ratio, p-tau181, p-tau181/Aβ42 ratio, p-tau217, and p-tau217/Aβ42 ratio. The vertical dashed line in each panel indicates the optimal single cutoff value determined using the Youden index. The solid black curve represents the predicted probability of Aβ PET positivity derived from a linear regression model. Colored density curves depict the distribution of biomarker values stratified by Aβ PET status, with gray shading indicating Aβ PET–negative participants and orange shading indicating Aβ PET–positive participants.

Abbreviations: Aβ, amyloid-β; PET, positron emission tomography; p-tau, phosphorylated tau.


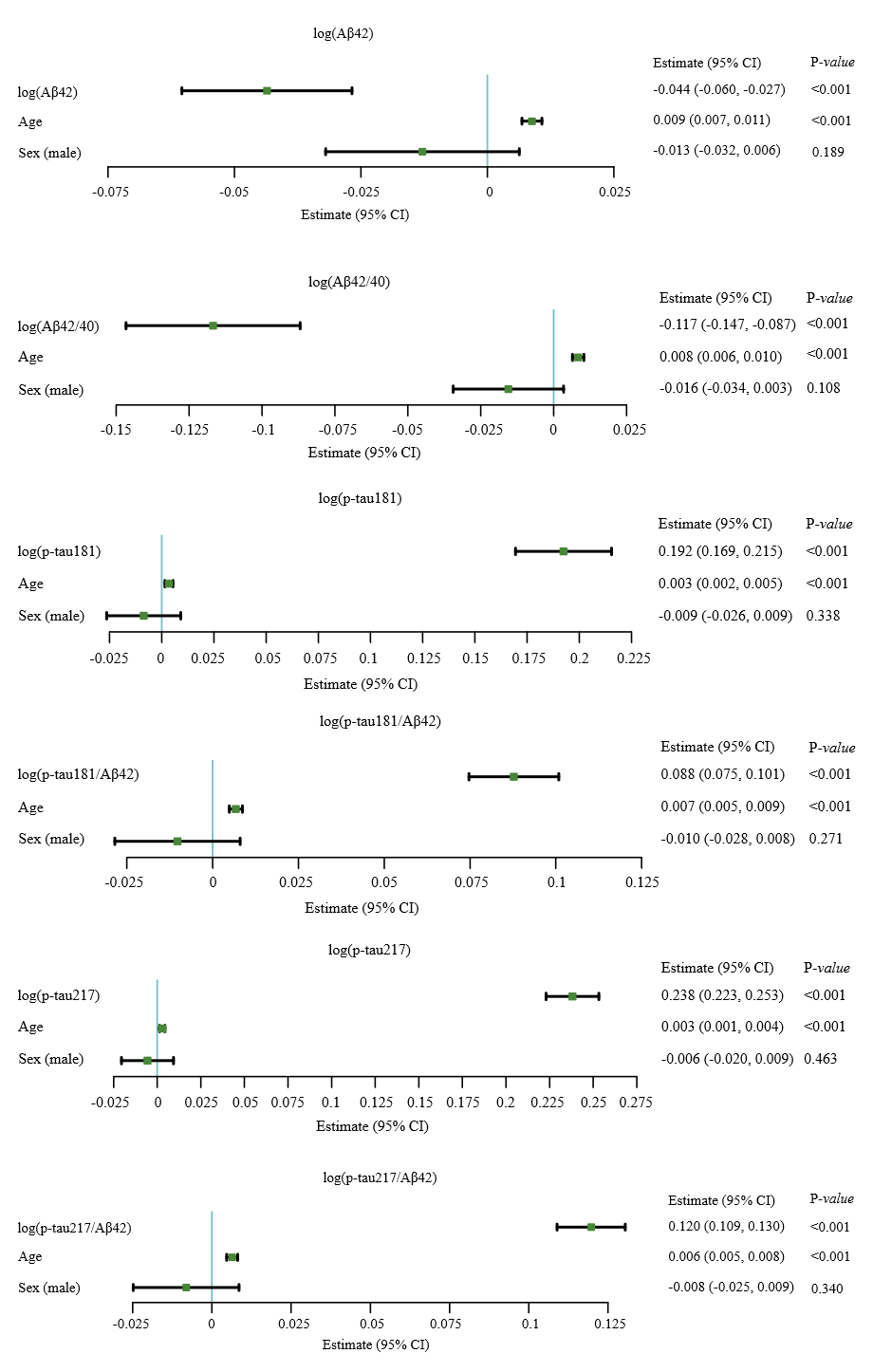
**Figure S2. Associations Between Plasma Biomarkers and Aβ-PET**

Estimates and 95% Cis from linear regression models including continuous measures of Aβ42, Aβ42/40, p-tau181, p-tau181/Aβ42, p-tau217, p-tau217/ Aβ42, as well as age and sex as predictors and Aβ-PET as outcome. Log-transformedand plasma biomarkers were used in all regression models.

**
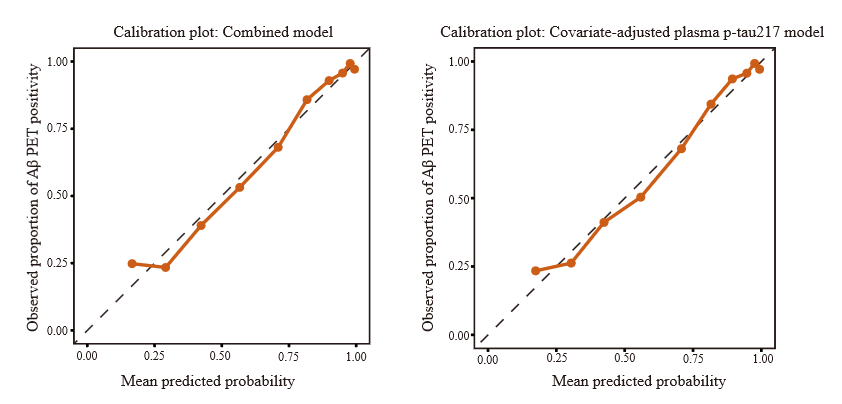
Figure S3. Calibration plots for the covariate-adjusted plasma p-tau217 model and the combined model for predicting Aβ PET positivity**

**Figure S4. Q-Q plots of residuals for linear regression models relating plasma biomarkers to continuous Aβ PET burden**
Q-Q plots of residuals from covariate-adjusted linear regression models assessing associations between six log-transformed plasma biomarkers and continuous Aβ PET SUVR. Models included age, sex, and APOE ε4 status as covariates. Panels show (A) plasma Aβ42, (B) plasma Aβ42/40 ratio, (C) plasma p-tau181, (D) plasma p-tau181/Aβ42 ratio, (E) plasma p-tau217, and (F) plasma p-tau217/Aβ42 ratio.

**Figure S5. Residuals-versus-fitted plots for linear regression models relating plasma biomarkers to continuous Aβ PET burden**
Residuals-versus-fitted plots from covariate-adjusted linear regression models assessing associations between six log-transformed plasma biomarkers and continuous Aβ PET SUVR. Models included age, sex, and APOE ε4 status as covariates. Panels show (A) plasma Aβ42, (B) plasma Aβ42/40 ratio, (C) plasma p-tau181, (D) plasma p-tau181/Aβ42 ratio, (E) plasma p-tau217, and (F) plasma p-tau217/Aβ42 ratio.


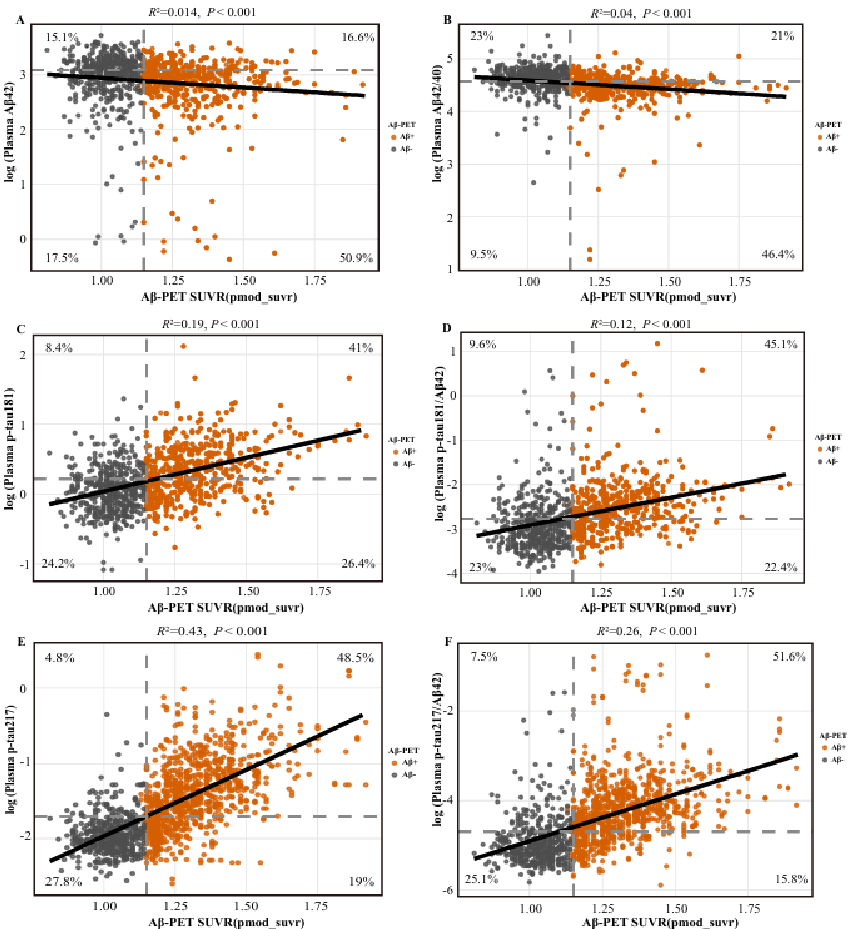


**Figure S6. Biomarker-SUVR plots with threshold overlays and quadrant percentages Scatterplots show the associations between six log-transformed plasma biomarkers and continuous Aβ PET SUVR. SUVR values were calculated using PMOD software.** Panels show (A) Aβ42, (B) Aβ42/40 ratio, (C) p-tau181, (D) p-tau181/Aβ42 ratio, (E) p-tau217, and (F) p-tau217/Aβ42 ratio. Black lines indicate linear regression fits shown for visual summary of the biomarker-SUVR associations, with the coefficient of determination (R²) and corresponding P values shown for each panel. Horizontal dashed lines indicate optimal biomarker cutoffs derived from ROC analyses using the Youden index to predict Aβ PET positivity; vertical dashed lines indicate the Aβ PET positivity threshold. Percentages indicate the proportion of participants within each quadrant defined by the two dashed lines, representing the proportions of true positives, true negatives, false positives, and false negatives. Orange dots represent Aβ PET–positive (Aβ+) participants, whereas gray dots represent Aβ PET–negative (Aβ–) participants.

Abbreviations: Aβ, amyloid-β; PET, positron emission tomography; p-tau, phosphorylated tau; ROC, receiver operating characteristic; SUVR, standardized uptake value ratio.

**Figure S**7**. Distribution of plasma biomarkers by amyloid status in the HABS-HD cohort**

**Figure S8. Associations between plasma biomarkers and amyloid PET burden (SUVR) in the HABS-HD cohort**

**Figure S9. Covariate-adjusted ROC curves and corresponding AUC-based model comparison in the HABS-HD cohort**

Receiver operating characteristic (ROC) curves for the classification of amyloid-β (Aβ) positivity using plasma biomarkers in the HABS-HD cohort. Models were adjusted for age, sex, and APOE ε4 status. The combined model included p-tau217, p-tau181, and Aβ42. The right panel presents a forest-style comparison of model performance, showing area under the curve (AUC) estimates with 95% confidence intervals for each model. Models are ordered by AUC, with the combined model highlighted. Consistent with the primary A4/LEARN analyses, p-tau217-based models demonstrated superior discrimination among individual biomarkers, while the combined model achieved the highest overall performance.
