# Supplementary material for: Plasma p-tau217, p-tau181, and Aβ42 predict amyloid PET positivity in cognitively unimpaired adults
Source: Alzheimers Res Ther. 2026 May 20;18:136. doi: 10.1186/s13195-026-02080-x (PMC13227887; doi:10.1186/s13195-026-02080-x)
Supplement: Supplementary file 2 — Supplementary Material 2. [file 13195_2026_2080_MOESM2_ESM.docx]

**Data Sharing Statement**

Data available: Yes

**Additional Information**

The A4 Study was a secondary prevention trial in preclinical Alzheimer's disease, aiming to slow cognitive decline associated with brain amyloid accumulation in clinically normal older individuals. The A4 Study was funded by a public-private-philanthropic partnership, including funding from the National Institutes of Health-National Institute on Aging, Eli Lilly and Company, Alzheimer's Association, Accelerating Medicines Partnership, GHR Foundation, an anonymous foundation, and additional private donors, with in-kind support from Avid Radiopharmaceuticals, Cogstate, Albert Einstein College of Medicine and the Foundation for Neurologic Diseases.The companion observational Longitudinal Evaluation of Amyloid Risk and Neurodegeneration (LEARN) Study was funded by the Alzheimer's Association and GHR Foundation. The A4 and LEARN Studies were led by Dr. Reisa Sperling at Brigham and Women's Hospital, Harvard Medical School, and Dr. Paul Aisen at the Alzheimer's Therapeutic Research Institute (ATRI) at the University of Southern California. The A4 and LEARN Studies were coordinated by ATRI at the University of Southern California, and the data are made available under the auspices of Alzheimer's Clinical Trial Consortium through the Global Research & Imaging Platform (GRIP). The complete A4 Study Team list is available on: https://www.actcinfo.org/a4-study-team-lists/. We would like to acknowledge the dedication of the study participants and their study partners who made the A4 and LEARN Studies possible.
